# Contrasting Symbiotic Patterns in Two Closely Related Lineages of Trimembered Lichens of the Genus *Peltigera*

**DOI:** 10.3389/fmicb.2018.02770

**Published:** 2018-11-16

**Authors:** Carlos José Pardo-De la Hoz, Nicolas Magain, François Lutzoni, Trevor Goward, Silvia Restrepo, Jolanta Miadlikowska

**Affiliations:** ^1^Departamento de Ciencias Biológicas, Universidad de Los Andes, Bogotá, Colombia; ^2^Department of Biology, Duke University, Durham, NC, United States; ^3^UBC Herbarium, Beaty Biodiversity Museum, University of British Columbia, Vancouver, BC, Canada

**Keywords:** *Coccomyxa*, *Nostoc*, sexual vs. asexual reproduction, species delimitation, symbiosis

## Abstract

Species circumscription is key to the characterization of patterns of specificity in symbiotic systems at a macroevolutionary scale. Here, a worldwide phylogenetic framework was used to assess the biodiversity and symbiotic patterns of association among partners in trimembered lichens from the genus *Peltigera*, section *Chloropeltigera*. We sequenced six loci of the main fungal partner and performed species discovery and validation analyses to establish putative species boundaries. Single locus phylogenies were used to establish the identity of both photobionts, *Nostoc* (cyanobacterium) and *Coccomyxa* (green alga). Distribution and specificity patterns were compared to the closely related clade, section *Peltidea*, which includes mainly *Peltigera* species with trimembered thalli. For section *Chloropeltigera*, eight fungal species (including five newly delimited putative species) were found in association with nine *Nostoc* phylogroups and two *Coccomyxa* species. In contrast, eight fungal species (including three newly delimited putative species) in section *Peltidea* were found in association with only four *Nostoc* phylogroups and the same two *Coccomyxa* species as for section *Chloropeltigera*. This difference in cyanobiont biodiversity between these two sections can potentially be explained by a significantly higher frequency of sexual reproductive structures in species from section *Chloropeltigera* compared to section *Peltidea*. Therefore, horizontal transmission of the cyanobiont might be more prevalent in *Chloropeltigera* species, while vertical transmission might be more common in *Peltidea* species. All *Peltigera* species in section *Chloropeltigera* are generalists in their association with *Nostoc* compared to more specialized *Peltigera* species in section *Peltidea*. Constrained distributions of *Peltigera* species that associate strictly with one species of green algae (*Coccomyxa subellipsoidea*) indicate that the availability of the green alga and the specificity of the interaction might be important factors limiting geographic ranges of trimembered *Peltigera*, in addition to constraints imposed by their interaction with *Nostoc* partners and by climatic factors.

## Introduction

Delimitation of fungal species is paramount to our understanding of the level of specificity among symbiotic partners in lichens and the processes shaping their evolutionary history (e.g., Magain and Sérusiaux, 2014; Magain et al., 2016). Improvements in analytical methods have changed the way systematists delimit species (e.g., Yang and Rannala, 2010; Lumbsch and Leavitt, 2011; Reid and Carstens, 2012; Zhang et al., 2013), and unveiled the existence of an increasing number of unrecognized and mostly cryptic fungal species (e.g., Weir et al., 2012; Sato et al., 2017). The general trend has been the recognition of a large number of lichen-forming species (ca. 20,000) found in association with a few photobionts, ca. 150 (Feuerer and Hawksworth, 2007; Honegger, 2009; Perez-Ortega et al., 2012; Feuerer, 2016). Recently, studies addressing the biodiversity of photobionts in selected lichens at various spatiotemporal scales (from a single thallus to lichen communities, and from a single species to species rich genera) have become increasingly popular (e.g., Muggia et al., 2010, 2013, 2014; Thüs et al., 2011; Dal Grande et al., 2012; Perez-Ortega et al., 2012; Leavitt et al., 2013, 2015; Ruprecht et al., 2014; Yahr et al., 2015; Hestmark et al., 2016; Magain et al., 2017a,b; Moya et al., 2017). Various degrees of specificity among partners have been found in lichens. Symbiont selectivity operates in two directions (i.e., mycobiont toward photobiont and vice versa) and factors driving these symbiotic associations vary at different spatial scales and are likely to vary at different temporal scales (Fedrowitz et al., 2012; Dal Grande et al., 2014; Muggia et al., 2014; Chagnon et al., 2018; Lu et al., 2018).

The lichen-forming genus *Peltigera* Wild (Peltigerales, Lecanoromycetes, Ascomycota) includes bimembered and trimembered species. The former are associations between mycobionts and cyanobacteria (cyanobionts) from the genus *Nostoc*, while the latter comprise these two partners and green algae (phycobionts) from the genus *Coccomyxa*. In trimembered thalli the phycobiont is the main photobiont, whereas *Nostoc* is restricted to external cephalodia. Molecular systematic studies of *Peltigera* confirmed and clarified established infrageneric phylogenetic relationships (e.g., Goffinet et al., 2003; Miadlikowska et al., 2003; Miadlikowska and Lutzoni, 2004; O’Brien et al., 2005), including the recent re-circumscription of broadly defined species, and exploration of reciprocal levels of specificity between mycobionts and cyanobionts (O’Brien et al., 2013; Miadlikowska et al., 2014; Magain et al., 2016, 2017a,b; Chagnon et al., 2018; Lu et al., 2018). Despite these efforts, phylogenetic affiliations among some sections of *Peltigera* remain uncertain (e.g., the monophyly of trimembered sections, and the placement of section *Hydrothyriae*) and species richness seems to be underestimated (e.g., within sections *Peltigera*, *Chloropeltigera* and *Horizontales*). While several studies addressed the identity and phylogenetic relationships of lichenized *Nostoc* (e.g., O’Brien et al., 2005, 2013; Otálora et al., 2010; Fedrowitz et al., 2011; Rikkinen, 2013; Magain and Sérusiaux, 2014; Miadlikowska et al., 2014; Magain et al., 2016), very little is known about the biodiversity of the symbiotic green alga *Coccomyxa* (Darienko et al., 2015; Malavasi et al., 2016).

Three *Peltigera* sections, i.e., *Chloropeltigera* (three known species: *P. leucophlebia, P. latiloba*, and *P. nigripunctata*)*, Peltidea* (five known species: *P. aphthosa*, *P.* *britannica*, *P. chionophila*, *P. frippii*, and *P. malacea*) and *Phlebia* (one known species: *P. venosa*), include all known trimembered *Peltigera* species and are composed almost exclusively of trimembered species (Miadlikowska and Lutzoni, 2000). According to Magain et al. (2017a) these three sections share a most recent common ancestor, but this relationship was not well supported. Most trimembered species of the genus *Peltigera* occur predominantly in the boreal biome with some exceptions where they can grow in lower latitudes but at high elevations (Martínez et al., 2003). Based on an ITS phylogeny, restricted to specimens collected in British Columbia, Canada, O’Brien et al. (2009) demonstrated that *P. leucophlebia* (section *Chloropeltigera*) includes at least three well-supported monophyletic groups, most likely representing putative cryptic species. To evaluate cyanobiont diversity associated with *Peltigera* thalli, strongly supported monophyletic groups (*Nostoc* phylogroups), serving as proxies for species, were circumscribed within a broad phylogenetic context of the genus *Nostoc* (O’Brien et al., 2013; Magain et al., 2017a). O’Brien et al. (2013) found five different *Nostoc* phylogroups (I, III, IV, V, and VI) in association with species of the three trimembered sections of *Peltigera*, each species showing a different degree of specificity toward *Nostoc* phylogroups. An unusually high level of reciprocal specificity between *Nostoc* and *P. malacea* was detected by O’Brien et al. (2013), which was confirmed by Miadlikowska et al. (2018) based on more specimens and additional loci. Based on a multi-locus phylogenetic study, Miadlikowska et al. (2018) also found that several mycobiont clades within section *Peltidea* could potentially represent new species. However, none of them were formally described due to the lack of diagnostic phenotypic features, the need for a more extensive sampling, and the need to assess the level of gene flow among these putative species. Therefore, the limited span of the phylogenetic, geographical, and molecular sampling of past studies, as well as the lack of information about the third partner (*Coccomyxa*), did not allow a comprehensive understanding of the spatiotemporal interactions among symbionts shaping trimembered *Peltigera* species.

Trimembered lichens represent an ideal system to study complex symbiotic interactions. Few studies have addressed symbiotic patterns of association in trimembered lichens within the context of robust phylogenies inferred for each of the three partners. The ancestral symbiotic state for the genus *Peltigera* is bimembered (one mycobiont in association with one cyanobiont), which is also the case for the entire order Peltigerales (Miadlikowska and Lutzoni, 2004). In other genera with bimembered and trimembered thalli (such as *Nephroma*), some evidence suggests that the evolutionary acquisition of a green algal partner by bimembered cyanolichens was accompanied by a change in cyanobiont composition (Lohtander et al., 2003; Fedrowitz et al., 2012). Specificity patterns toward *Nostoc* have also been hypothesized to be different between bimembered and trimembered species, the latter being associated with a narrower set of *Nostoc* lineages (Elvebakk et al., 2008). Also, the mode of reproduction (sexual vs. asexual) of the lichen-forming fungus is linked to the type of photobiont transmission from one generation to the next (horizontal vs. vertical, respectively), which can be an important factor driving symbiotic specificity. Sexually reproducing fungal species must reacquire their photobiont at each generation, favoring generalists, whereas asexually reproducing species promote vertical transmission of their photobionts, which is likely to result in high reciprocal specificity (Paulsrud et al., 1998; Otálora et al., 2010; Dal Grande et al., 2012; Hestmark et al., 2016; Magain et al., 2017a).

In this study we assembled a multilocus dataset for *Peltigera* species, and a single locus dataset for each of the photosynthetic partners (*Nostoc* and *Coccomyxa*), from section *Chloropeltigera* (and for selected representatives from section *Peltidea*), to generate a worldwide phylogeny for this section. We discuss the potential contribution of various biotic and abiotic factors (i.e., symbiont distribution, diversification rates, and photobiont transmission) in shaping the spatial and evolutionary patterns of symbiotic association and levels of specificity among the three main partners of these lichens.

## Materials and Methods

### Taxon Sampling

Ninety-two specimens from section *Chloropeltigera* were chosen across its global distribution (mostly the boreal biome) for this study. These individuals were selected from herbarium material and collected by the authors and collaborators during fieldtrips in China, Russia, Norway, Iceland, Canada, and the United States. Additionally, 32 specimens from section *Peltidea* used in Miadlikowska et al. (2018) were also included. Supplementary Table S1 summarizes the voucher information for each specimen used.

### Molecular Data Acquisition

Total DNA was extracted from fresh and herbarium specimens lacking visual symptoms of fungal infections. We followed a modified protocol from Zolan and Pukkila (1986) using 2% sodium dodecyl sulphate (SDS) as extraction buffer. In order to get sufficient amounts of DNA for cyanobionts, several cephalodia were collected from multiple thallus lobes. For the mycobiont, we sequenced six loci, including one nuclear ribosomal locus: the Internal Transcribed Spacer (ITS) using primers ITS1F and ITS4 (White et al., 1990); two protein coding genes: β-*tubulin* (β-*tub*) using primers bt_34F (O’Brien et al., 2009) and BT2B (Glass and Donaldson, 1995), and the RNA Polymerase II largest subunit (*RPB1*) using primers RPB1-AF (Stiller and Hall, 1997) and RPB1-CR (Matheny et al., 2002); and the non-coding part of three Collinear Orthologous Regions (COR) that were recently selected based on genomic synteny analyses: COR1b using primers COR-1bF and COR-1bR-B, COR3 using primers COR-3F-A and COR-3R-B, and COR16 with primers COR-16Fout and COR-16Rmid1 (Magain et al., 2017b). To amplify the three COR markers we followed the PCR protocol included in Magain et al. (2017b), whereas for the remaining loci we used the conditions described in Miadlikowska et al. (2014).

The *rbcLX* locus of *Nostoc* (comprised of the last 82 amino acids of the RUBISCO large subunit, a putative chaperone gene [*rbcX*] and two intergenic spacers) was amplified using primers CX and CW (Rudi et al., 1998) following the protocol of O’Brien et al. (2013). *Cocccomyxa*-specific primers were designed to target the ITS region of this algal photobiont using DNA isolated from the entire thallus (ITSCOF: 5′ GAC GGA GAT TTT CAA GTT GG 3′; ITSCOR: 5′ CCT CCC ACC TAG AGG AAG G 3′). The following PCR settings were used: 94°C for 1 min; 35 cycles of 94°C for 30 s, 55°C for 30 s and 72°C for 1 min; and a final elongation at 72°C for 10 min.

All PCR reactions were carried out in a final volume of 25 μL containing 2.5 μL of buffer, 2.5 μL of 10 mM dNTPs, 1.25 μL of 10 mM primers, 0.15 μL of TargeTaq^TM^ (Southern BioLabs, Cary, NC, United States), 2.5 μL of BSA, 15 μL of double-sterilized ultrapure H_2_O and 1 μL of DNA. All PCR products were cleaned and sequenced as described in Miadlikowska et al. (2014). All sequences were assembled using Geneious v. 7.1. (Drummond et al., 2011). GenBank accession numbers are listed in Supplementary Table S1.

### Multiple Sequence Alignments and Datasets

All sequences were subjected to BLAST searches using GenBank database to confirm their expected identity. Multiple sequence alignments were generated for each locus using the MAFFT v. 7 (Katoh and Standley, 2013) online platform^1^ with the Q-INS-i strategy. Alignments were then adjusted manually using MacClade v. 4.08 (Maddison and Maddison, 2005) and Mesquite v. 2.75 (Maddison and Maddison, 2010). Manually delimited ambiguously aligned regions (following Lutzoni et al., 2000) were excluded from subsequent phylogenetic analyses. Single locus maximum likelihood phylogenetic analyses were performed for the mycobiont to detect topological conflicts with a 70% bootstrap threshold using the Hypha package (Oliver et al., 2013) as implemented in Mesquite v. 2.75.

As no significant conflicts were found between single-locus phylogenies, four datasets were assembled: (1) a mycobiont concatenated matrix with 42 ingroup taxa comprising all specimens from section *Chloropeltigera* with at least four of the six targeted loci, and eight outgroup taxa from sections *Phlebia* and *Peltidea*; (2) a *Nostoc* matrix with a total of 425 *rbcLX* sequences representing a broad sampling of published symbiotic and free-living *Nostoc* from *Nostoc* Clade I, and Clade II subclades 1, 2, and 3 (*sensu* Magain et al., 2017a), including 54 new sequences generated for this study and 28 from Miadlikowska et al. (2018); (3) a *Coccomyxa* matrix consisting of 81 new ITS sequences generated as part of this study and 70 sequences from the latest systematic revision of this genus (Malavasi et al., 2016), including both free-living and lichenized *Coccomyxa* found in association with Ascomycota (ascolichens) and Basidiomycota (basidiolichens); and (4) a concatenated matrix for single representatives of each of the 17 species from sections *Chloropeltigera, Peltidea* and *Phlebia* for which sequences from the same four loci were available (ITS, *β-tubulin*, COR1b and COR3). Perl scripts “compare_and_choose.pl,” “adapt_from_file.pl,” and “exclude_and_adapt.pl” (Magain, unpublished) were used to prepare the concatenated matrices used in this study. All alignments and phylogenies were deposited in TreeBASE^2^ (Pardo-De la Hoz et al., 2018).

### Phylogenetic Analyses

PartitionFinder V.1.1.1 (Lanfear et al., 2012) was used to find the best partition scheme and model of nucleotide substitution for both single and multilocus datasets using the rcluster search algorithm and the corrected Akaike Information Criterion (AICc). The search was performed with the following starting subsets: (1) ITS1, 5.8S, ITS2, β-*tubulin* 1st, 2nd, 3rd codon positions and introns, *RPB1* 1st, 2nd, 3rd codon positions and intron, COR1b, COR3 and COR16 for the concatenated mycobiont matrix; (2) *rbcL* 1st, 2nd, 3rd codon positions and *rbcX* 1st, 2nd, 3rd codon positions for the *Nostoc* matrix; (3) ITS1, 5.8S and ITS2 for the *Coccomyxa* matrix; and (4) ITS1, 5.8S, ITS2, β-*tubulin* 1st, 2nd, 3rd codon positions and introns, COR1b and COR3 for the matrix covering three sections of the genus *Peltigera*. Maximum likelihood (ML) searches for the most likely tree and bootstrap replicates (1000) were performed on each dataset using RAxML HPC2 v. 8 (Stamatakis et al., 2008; Stamatakis, 2014) as implemented in CIPRES (Miller et al., 2010) using the GTRGAMMA model estimated for each data subset.

### Mycobiont Species Delimitation

We coupled species discovery and validation methods to delimitate mycobiont species (Leavitt et al., 2015; Magain et al., 2017b). For section *Chloropeltigera*, we used the ML tree estimated based on the concatenated mycobiont matrix to infer putative species boundaries with the Maximum Likelihood Poisson Tree Process (PTP; Zhang et al., 2013) as implemented on the web server^3^. We generated single locus chronograms using an uncorrelated lognormal relaxed clock in BEAST v 1.8.3 (Drummond and Rambaut, 2007; Drummond et al., 2013) with default priors. We performed two independent BEAST searches with four chains each. Both searches were run for 10,000,000 generations sampling every 1000th generation for a total sample size of 20,000 trees. Effective sample size for the estimated parameters and convergence was assessed in Tracer v 1.6 (Rambaut and Drummond, 2013). For each locus, a set of 500 trees from the estimated posterior distribution were subsampled using LogCombiner v. 1.8.2 (Rambaut and Drummond, 2015) after discarding 10% of the samples as burnin. These sets of trees were used to infer single locus-based species delimitations using a bayesian implementation of the Generalized Mixed Yule Coalescent model (bGMYC) (Reid and Carstens, 2012). The bGMYC analyses were run for 50,000 generations with threshold values of 2 and 25 and a starting point of “1,1,25.” The thinning parameter was set to 100 and the burnin to 10,000 samples. We assigned individuals to putative species when the probability of grouping haplotypes together was higher than the probability of all alternative groupings that included at least one haplotype from this putative species.

The most splitting delimitation scheme inferred with the discovery methods was set as prior for species validation in Bayesian Phylogenetics and Phylogeography (BP&P) (Rannala and Yang, 2003; Yang and Rannala, 2010) using the concatenated mycobiont matrix. The gamma distribution parameter priors for τ and θ were set to G(2, 1000) and G(2, 100). The analysis was run for 1,000,000 generations sampling every 10th generation and discarding the first 10% as burnin. Daughter lineages of nodes with posterior probabilities of speciation ≥0.95 were considered well-supported species. An additional bGMYC analysis was run on a matrix combining our mycobiont ITS dataset with the ITS sequences from section *Peltidea* generated by Miadlikowska et al. (2018). The purpose of this analysis was to generate a species delimitation scheme within a phylogenetic framework that included all *Peltigera* sections with trimembered taxa. This uniform approach to species delimitation across these three sections enabled inter-section comparisons of specificity and mode of reproduction (sexual vs. asexual) for all known trimembered *Peltigera* lichens.

### Symbiont Specificity Assessment

To account for the differences in cyanobiont sample sizes between mycobiont species, a rarefaction analysis was performed to estimate the distribution of the number of *Nostoc* phylogroups as a function of the number of samples per section (*Peltidea* and *Chloropeltigera*). The R package iNEXT v. 1.0 (Hsieh et al., 2013) was used for the rarefaction analysis with 50 bootstrap replicates to generate a 95% confidence interval.

### Quantification of Sexual and Asexual Reproduction

We examined 253 well-preserved specimens (116 sequenced in this study and listed in Supplementary Table S1; 137 from Miadlikowska et al., 2018) and scored them for the presence or absence of apothecia as a proxy for sexual vs. asexual reproduction. ITS haplotype diversity (*H*) was computed for each species using equation 1.

(1) $$H=\frac{N}{N-1}\left(1-\sum_{\text{i}}x_{\text{i}}^{2}\right)(\mathrm{Nei and Tajima},\;1981)$$

where *x*_i_ is the relative frequency of each haplotype in the sample and *N* is the total sample size (i.e., the number of haplotypes from each species). A linear regression and a statistical correlation test were done in R considering all species with more than 10 available samples. We used the R package iNEXT for the estimation of the rarefaction curves and for comparing the distribution of the number of ITS haplotypes as a function of the sample size per section between *Chloropeltigera* and *Peltidea.* Fifty bootstrap replicates were completed to generate a 95% confidence interval.

## Results

### Mycobiont Phylogeny and Species Delimitation

**FIGURE 1:**
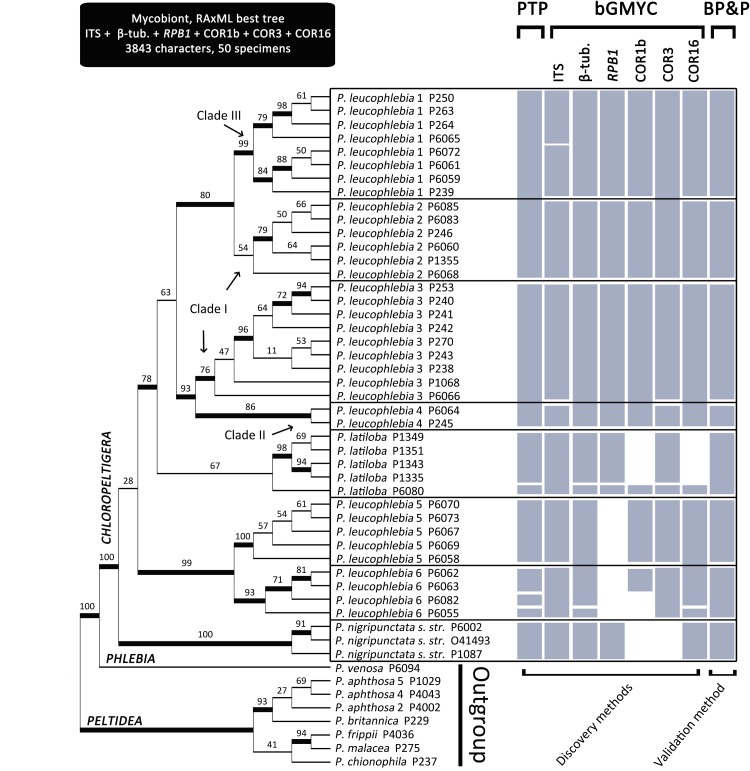
Maximum likelihood phylogeny of *Peltigera* section *Chloropeltigera* (mycobiont) based on six combined loci. Species were delimited by implementing discovery (PTP and bGMYC) and validation (BP&P) methods on 42 specimens from section *Chloropeltigera.* Representatives of sections *Phlebia* and *Peltidea* were used as outgroup (Magain et al., 2017a). Numbers above branches are bootstrap proportions and thick branches represent nodes with bootstrap values ≥70%. Gray bars represent species delimitation scheme inferred by each method. Boxes indicate species delimitation based on BP&P. Arrows indicate the three putative cryptic clades within *P. leucophlebia* s. lat. reported in O’Brien et al. (2009).

Based on the BP&P species validation method, section *Chloropeltigera* comprised eight well-supported lineages (i.e., speciation posterior probability ≥0.95), which might represent species (Figure 1). *P. leucophlebia* *sensu lato* was recovered as non-monophyletic because it comprised *P. latiloba* (Figure 1 and Supplementary Figure S1). Most analyses split *P. leucophlebia s. l.* (Figure 1) into five to six distinct lineages. However, a few conflicts were observed between the species delimitation schemes inferred with the discovery methods and their validation with BP&P (e.g., the split between *P. leucophlebia* 3 and *P. leucophlebia* 4). Newly delimited lineages within *P. leucophlebia s. l.* were well supported (bootstrap values above 75%), except for the monophyly of *P. leucophlebia* 2, which received a bootstrap support of 54% (Figure 1 and Supplementary Figure S1). The monophyly of *P. latiloba* was recovered with bootstrap support of 67% and was nested inside *P. leucophlebia s. l.* (Figure 1 and Supplementary Figure S1). Four of the discovery analyses split this species into two distinct lineages, i.e., specimen P6080 represents one lineage distinguishable from the remaining individuals that form a well-supported clade. However, BP&P merged these two lineages into one (pp = 0.96; Figure 1). The bGMYC analysis performed on the ITS matrix for sections *Chloropeltigera* and *Peltidea* (combined) delimited the same species as the BP&P analysis restricted to section *Chloropeltigera* (result not shown).

### Photobiont Identity

**FIGURE 2:**
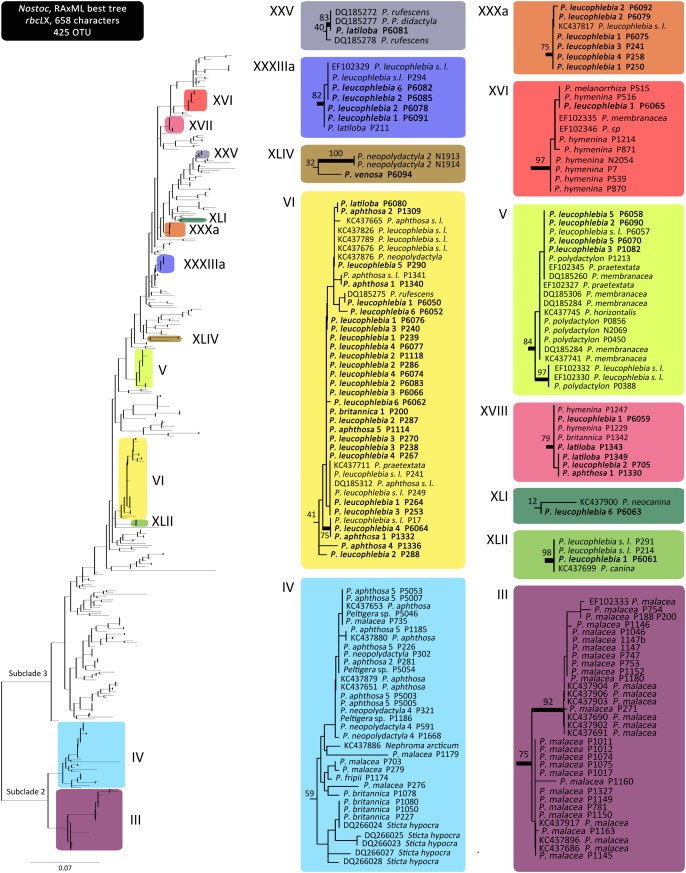
*Nostoc rbcLX* maximum likelihood phylogeny. Clades highlighted with colors on the complete tree, without tip labels, on the left, correspond to the phylogroups associated with *Peltigera* species included in this study. Detailed tip labels are shown on the clades on the right. Top left Roman numerals correspond to the *Nostoc* phylogroup numbering system initiated by O’Brien et al. (2013) and further developed by Magain et al. (2017a). Tip label names correspond to the mycobiont forming the thallus within which the *Nostoc* strain was sequenced. Names in bold correspond to the sequences generated in this study. Thick branches represent bootstrap support ≥70%.

Eighty *Nostoc rbcLX* sequences, obtained from the three sections of the genus *Peltigera* with trimembered lichens, clustered in 12 monophyletic groups (*Nostoc* phylogroups) in the ML tree (Figure 2), following phylogroups designation *sensu* Magain et al. (2017a). Seven of these phylogroups had strong bootstrap support (≥70%; Figure 2). Two of these phylogroups (III and IV) are part of *Nostoc* clade 2 subclade 2 *sensu* Otálora et al. (2010), while the remaining phylogroups belong to *Nostoc* clade 2 subclade 3 (Figure 2). Three sequences that could not be assigned to any known phylogroup (i.e., phylogenetically placed outside existing and newly defined phylogroups) were labeled using an *rbcLX* unique haplotype number (Supplementary Table S1).

**FIGURE 3:**
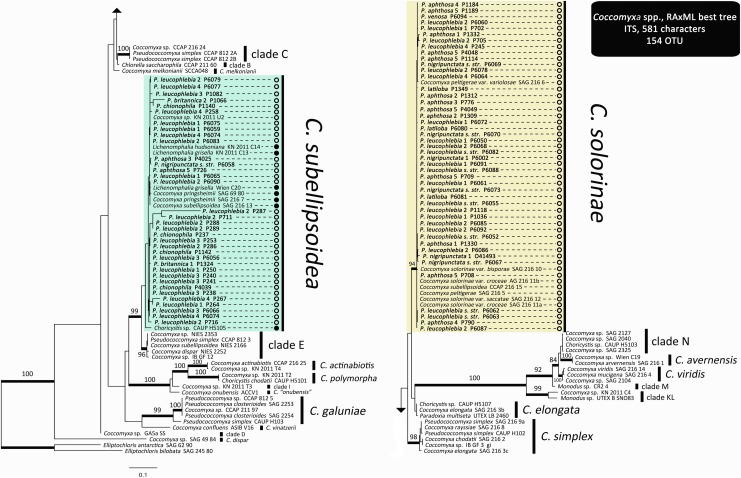
*Coccomyxa* ITS maximum likelihood phylogeny. Thick branches represent ≥70% bootstrap support. Labeled clades correspond to species delimited by Malavasi et al. (2016). Clades highlighted with colors correspond to species found in association with trimembered *Peltigera* species. Names in bold correspond to the sequences generated for this study and refer to the mycobionts forming the thalli containing the green alga (phycobiont) *Coccomyxa*. Open circles, Ascomycota mycobiont; Closed circles, Basidiomycota mycobiont.

The ITS phylogeny for *Coccomyxa* (Figure 3) revealed the same major clades and all species-level phylogenetic relationships reported by Malavasi et al. (2016). Newly added sequences from trimembered *Peltigera* species fell inside two strongly supported species: *C. solorinae* and *C. subellipsoidea.* The latter clade comprises photobionts associated with lichen-forming fungi from both Ascomycota and Basidiomycota phyla, whereas *C. solorinae* has been found only in association with Ascomycota fungi (Figure 3).

### Symbiotic Specificity and Geographic Patterns

**FIGURE 4:**
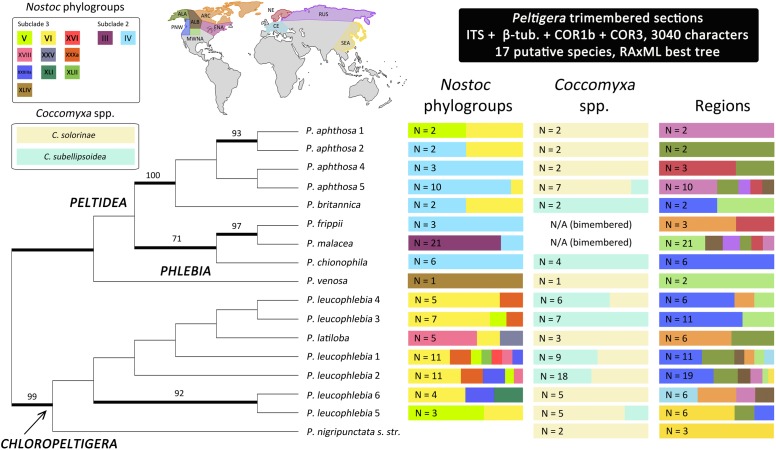
Unrooted maximum likelihood phylogenetic relationships among the three *Peltigera* sections that include trimembered thalli. Thick branches represent ≥70% bootstrap support. Bars on the right indicate the proportion of *Peltigera* specimens associating with *Nostoc* phylogroups, *Coccomyxa* species, and inhabiting various geographic regions. Only specimens with sequence data from at least one photobiont were considered when calculating these proportions. We divided the distribution ranges of our samples into ten broad geographic regions based on latitudinal, oceanic and orographic conditions: Alaska (ALA), Alberta (ALB), Arctic (ARC), Central Europe (CE), Eastern North America (ENA), Mid-Western North America (MWNA), Northern Europe (NE), Pacific North West (PNW), Russia (RUS), and South East Asia (SEA). The Assigned regions for each specimen are listed in Supplementary Table S1. N, Sample size; N/A, Not applicable.

*Peltigera* species from section *Chloropeltigera* were found in association with nine *Nostoc* phylogroups, each species associating with up to seven phylogroups (no data were available for *P. nigripunctata s. str.*) (Figure 4). Species in section *Peltidea* were found in association with four phylogroups, each species associating with no more than two phylogroups. The cyanobiont from the only individual of *P. venosa* (section *Phlebia*) studied by us was identified as a rare *Nostoc* phylogroup (XLIV). Phylogroups (III and IV) from *Nostoc* clade 2 subclade 2 (Figure 2) are associated exclusively with *Peltigera* species from section *Peltidea*, whereas phylogroups from *Nostoc* clade 2 subclade 3 were found in association with species from all three sections (*Chloropeltigera, Phlebia* and *Peltidea*).

Both *Coccomyxa solorinae* and *C. subellipsoidea* were present in all three *Peltigera* sections without any obvious patterns of specificity toward a single fungus or cyanobiont (Figure 4). However, some mycobiont species showed preference for one of the two *Coccomyxa* species, e.g., *P. leucophlebia* 3 was always found with *C. subellipsoidea*, whereas *P. leucophlebia* 6 was found with *C. solorinae*.

In general, *Peltigera* species within these three sections, and their *Nostoc* phylogroups, have wide geographic distributions, except *P. chionophila* and *P. nigripunctata s. str.* 1 which are restricted to the Pacific North West (PNW) and South East Asia (SEA), respectively (Figure 4 and Supplementary Table S1). *Coccomyxa solorinae* was present in every sampled region whereas *C. subellipsoidea* was only found in North America (Supplementary Table S1).

### Proportion of Sexual vs. Asexual Reproduction

**FIGURE 5:**
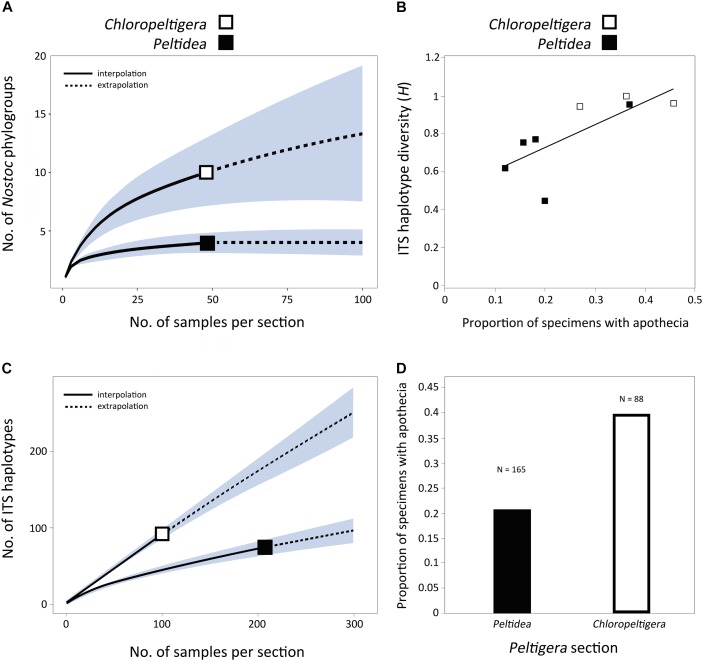
Assessment of specificity and reproductive mode in sections *Chloropeltigera* and *Peltidea.* **(A)** Rarefaction curve showing the distribution of the number of *Nostoc* phylogroups as a function of the number of samples per section. Shaded areas represent the 95% confidence intervals. **(B)** Correlation between ITS haplotype diversity and frequency of occurrence of apothecia. Each square represents a species. Only species with more than 20 specimens were considered for this analysis. Correlation test *p* < 0.05; *R*^2^ = 0.5554. **(C)** Rarefaction curve showing the distribution of the number if ITS haplotypes as a function of the number of samples per section. Shaded areas represent the 95% confidence intervals. **(D)** Frequencies of specimens with apothecia in sections *Chloropeltigera* and *Peltidea.*

A significant positive correlation (*p* < 0.05) was found between the proportion of specimens with apothecia and ITS haplotype diversity (*H*) in species from sections *Chloropeltigera* and *Peltidea* (Figure 5B). There were twice as many ITS haplotypes in section *Chloropeltigera* as in section *Peltidea* when accounting for sample sizes (Figure 5C). The proportion of specimens with apothecia in section *Chloropeltigera* (0.39) was nearly twice as large as for section *Peltidea* (0.21; Figure 5D). In general, species from section *Chloropeltigera* showed higher ITS haplotype diversity and higher proportion of specimens with sexual structures (apothecia) than most species from section *Peltidea* (Figure 5B).

## Discussion

### Mycobiont Species Delimitation

Previous studies have already shown the putative cryptic biodiversity within section *Chloropeltigera* (O’Brien et al., 2009). Based on ITS sequences from specimens collected in British Columbia, three well-supported clades were identified within *P. leucophlebia s. l.*, however, no morphological characters to recognize them as separate species were provided. Using a broader molecular and geographical sampling, we confirm two (Clade II and Clade III) of these lineages within this section (Figure 1). Together with the three currently recognized species (*P. leucophlebia, P. latiloba* and *P. nigripunctata*) (Holtan-Hartwig, 1993, 2005; Vitikainen, 1994), section *Chloropeltigera* contains at least eight putative species, five of which are new and represent *P. leucophlebia* *s. l*. A more extensive sampling might reveal that *P. latiloba* represents two species (P6080 was recognized by PTP and bGMYC as a different putative species). These results can serve as a starting point for a study designed to assess gene flow. The apparent lack of distinguishing phenotypic traits among these putative species could result from gene flow and may be reflected in species population dynamics.

### Photobiont Identity, Distributions, and Switches

We found 12 *Nostoc* phylogroups (*sensu* Magain et al., 2017a) consorting with *Peltigera* in the three sections *Chloropeltigera, Pelidea*, and *Phlebia* (Figure 2). Phylogroups III, IV, V, and VI had already been reported to consort with trimembered *Peltigera* species (O’Brien et al., 2013). In that study, one specimen of *P. britannica* (section *Peltidea*) consorted with *Nostoc* phylogroup I, a finding not confirmed by us perhaps due to low sampling. The remaining associations between the mycobiont species and *Nostoc* phylogroups reported by O’Brien et al. (2013) from sections *Chloropeltigera* and *Peltidea* were confirmed and expanded by our study. Phylogenetic branching and very short internodes recovered for most of the phylogroups suggest a predominantly clonal population structure (Figure 2), as reported previously for *Peltigera*-associated *Nostoc*, including from trimembered sections by O’Brien et al. (2013), and from several cyanolichen genera (Wirtz et al., 2003). This pattern is consistent with the hypothesis that recombination among the symbiotic *Nostoc* is not frequent in nature, however, in contrast with other studies which reported phylogenetic incongruence among different loci sequenced for these cyanobionts (O’Brien et al., 2005; Kaasalainen et al., 2012).

The primers designed for this study allowed culture-independent amplification of the ITS locus for *Coccomyxa* using metagenomic DNA extracted from lichen thalli. Our phylogeny for *Coccomyxa* is consistent with the hypothesis that lichenization occurred twice during the evolutionary history of this genus (Figure 3). So far, *C. solorinae* has been found only in two sister lichen genera, *Peltigera* and *Solorina* (Peltigeraceae, Ascomycota), while *C. subellipsoidea*, previously reported only from the basidiolichen genus *Lichenomphalia* (Zoller and Lutzoni, 2003; Muggia et al., 2011; Yahr et al., 2015), is now known to consort also with *Peltigera* (Figure 3). We conclude that there is no reciprocal specificity between these algal species and members of these two fungal phyla. Moreover, we found substantially less biodiversity in *Coccomyxa* (two species: *C. solorinae* and *C. subellipsoidea*) than in *Nostoc* (12 phylogroups) associated with 17 putative *Peltigera* species in these three sections (Figure 4).

Most of the putative *Peltigera* species included in this study have wide distributions across the boreal biome. In contrast, *P. chionophila* and *P. leucophlebia* 3 seem to be restricted to North America and specifically associate with *C. subellipsoidea* (Figure 4), which was only found in North America (Figure 4 and Supplementary Table S1). Because *C. subellipsoidea* can also associate with other *Peltigera* species with wider geographic distributions, *P. chionophila* and *P. leucophlebia* 3 may represent cases where the distribution of the photobiont limits the geographic range of the mycobiont (Louthan et al., 2015). *P. chionophila* is restricted to regions with a heavy, prolonged snow cover (Goward and Goffinet, 2000). The signatures of adaptation to cold environments found in the *C. subellipsoidea* genome by Blanc et al. (2012) could indicate that this alga plays a role in the snow tolerance observed in *P. chionophila.* However, because *C. subellipsoidea* is found in association with several other lichenized fungi, which grow in broader environmental conditions (e.g., *P. leucophlebia* 3, *Lichenomphalia* spp.), it is likely that additional factors are related to the snow tolerance displayed by *P. chionophila.* It was previously demonstrated that photobiont switches and associations with a higher number of photobionts (generalist pattern of association for a mycobiont species) can be associated with the colonization of new niches and geographic regions by the mycobionts, followed by a subsequent increase in specificity (specialist pattern of association) (Fernández-Mendoza et al., 2011; Magain et al., 2017a). However, some aspects of these patterns of association are spatially scale-dependent (Chagnon et al., 2018; Lu et al., 2018). Within the boreal biome, climatic factors are more limiting for some mycobionts than the availability of their photobiont, as was recently demonstrated for a few *Peltigera* species from section *Polydactylon* across the boreal zone in Québec, Canada (Lu et al., 2018). Therefore, these photobiont switches are perhaps more likely at an interbiome scale, or its equivalent along an altitudinal gradient, and involves mycobiont species with distributions covering more than one biome. Previously reported shifts in lichen-associated algal communities (*Trebouxia* photobionts of *Lecanora rupicula* and *Lasallia*) along altitudinal gradients (Blaha et al., 2006; Dal Grande et al., 2018) support the hypothesis that the abiotic environment may act as filter for both partners of the symbiosis, hence shaping their partnership as well as geographic ranges. Constrained distributions can also result from increased symbiotic specificity (Magain et al., 2017a). This can be the product of ancient coevolutionary histories (Thompson, 2005; Magain et al., 2017a) coupled with interspecies signaling needed to reconstitute the lichen symbiosis when the photobiont is transmitted horizontally (Etges and Ott, 2001).

Most *Peltigera* species form bimembered thalli with *Nostoc* as their only photobiont, and they can be found in all biomes (Martínez et al., 2003). However, the mainly boreal distributions of trimembered *Peltigera* species (so far not reported from the Southern Hemisphere) seem limited by the ranges of the green alga *Coccomyxa*, which appears to be adapted to cold environments (Blanc et al., 2012). In contrast, lichenized *Nostoc* associated with *Peltigera* are widespread across vast geographic regions and, therefore, are less likely to limit the distribution of *Peltigera* species (Lu et al., 2018). Several *Nostoc* phylogroups associated with the three *Peltigera* sections of trimembered species (e.g., phylogroup V) are known to associate with *Peltigera* species from other sections, which also occur outside of the boreal biome (O’Brien et al., 2013; Magain et al., 2016). A previous study suggested that cyanobiont composition has evolved differently in bimembered and trimembered lichens (Lohtander et al., 2003). However, this seems unlikely as several phylogroups are shared between bi- and trimembered *Peltigera* species from different sections, as well as mycobionts from other genera within the order Peltigerales (Figure 2).

### Evolution of Specificity in Sections *Chloropeltigera* and *Peltidea*

Two contrasting patterns of mycobiont specificity toward their *Nostoc* partners were observed in sections *Chloropeltigera* and *Peltidea* (Figure 4). With the same number of putative species in both sections, mycobionts from *Chloropeltigera* associate with nine different phylogroups of *Nostoc*, whereas mycobionts from *Peltidea* with only four. Moreover, no putative species in section *Peltidea* was found to associate with more than two *Nostoc* phylogroups, while in section *Chloropeltigera*, one putative species was associated with seven different *Nostoc* phylogroups (Figure 4). The occurrence of generalist trimembered *Peltigera* species in section *Chloropeltigera* does not support the hypothesis that trimembered lichen-forming fungi tend to be more specialized than bimembered species as suggested by Elvebakk et al. (2008) in the family Pannariaceae. Specificity of lichen-forming fungi and their photobionts seems to be clade dependent. For example, our study shows that some members from section *Peltidea* as well as their *Nostoc* partners (from *Nostoc* subclade 2) are highly specific toward each other, confirming, based on a broader geographic sampling, high reciprocal specificity between *Nostoc* phylogroup III and *P. malacea* (O’Brien et al., 2013; Miadlikowska et al., 2018). Previous studies suggest that geographic and ecological factors can drive the differences in the specificity of the association in both cyano- and chlorolichens (Fedrowitz et al., 2012; Muggia et al., 2014). Overall, cases of one-to-one specificity are very rare in lichens. Some exceptions to this trend include two *Peltigera* species from section *Polydactylon* (*P. neopolydactyla* 5 and *P*. sp. 11 associated with phylogroups XIb and IX, respectively) reported by Magain et al. (2017a), and five species of gelatinous lichens in the *Collemataceae* (Otálora et al., 2010). Usually, mycobiont species are specialized on a few *Nostoc* lineages, and *Nostoc* phylogroups associate with a broader set of mycobiont species (Myllys et al., 2007; Magain et al., 2017a; but see Blaha et al., 2006). The uniqueness of the specificity pattern detected in *P. malacea* might indicate that reversion to a bimembered thallus (Miadlikowska and Lutzoni, 2004; Figure 4) evolved not only through the loss of the green alga *Coccomyxa* but also through replacement of the ancestral cyanobiont(s) by a *Nostoc* from phylogroup III (Figure 2). The overall observed association pattern might indicate that a transition to a symbiotic association with *Nostoc* subclade 2 could have driven the specialization of *Peltigera* species in section *Peltidea* (Figure 4).

The relatively small number of individuals sampled from some species in section *Peltidea* (Figure 4) make it difficult to establish whether they are specialist or non-specialist (Magain et al., 2017b). To get around this, we assessed the degree of specificity by comparing the distribution of the number of *Nostoc* phylogroups as a function of the number of samples at the section level. Our results support previous insights on cyanobiont specificity reported from selected lineages in section *Peltidea* (Paulsrud et al., 1998; O’Brien et al., 2013) and show that species in section *Chloropeltigera* are generalists, compared to species in section *Peltidea*, in their association with *Nostoc* phylogroups (Figure 5A).

Several mechanisms have been proposed to explain differences in specificity in lichens. Differences in rate of mycobiont diversification have been shown to affect specificity in *Peltigera* section *Polydactylon* (Magain et al., 2017a). However, no significant rate shifts were found among trimembered lineages (data not shown). Limited geographic distribution and photobiont availability may also be a factor (Dal Grande et al., 2014), but as outlined earlier, most mycobionts and cyanobionts are widespread across the boreal biome. Also, there seems to be a preference for different photobiont lineages at the section level, with *Peltidea* associating mainly with *Nostoc* from subclade 2, and *Chloropeltigera* only associating with *Nostoc* from subclade 3 (Figure 4). These preferential interactions found in trimembered sections are in agreement with an anti-nested pattern of association detected with ecological networks analyses to assess the structure of symbiotic interactions in section *Polydactylon* at a global scale (Chagnon et al., 2018). For comparison, bimembered lichen-forming fungi from the genus *Protoparmelia*, associated with green algae (*Trebouxia* spp.) have been shown to vary in their specificity depending on macroclimatic variables (Singh et al., 2017). Although the *Peltigera* species studied here generally do not occupy geographic regions with contrasting macroclimates, it is possible that microclimatic differences exist within the distributions of species from different sections, and that such differences might affect symbiont availability and the association patterns. However, a co-occurrence study of the genus *Peltigera* and their *Nostoc* partners along a longitudinal and latitudinal transect of nearly 1300 km each, in the boreal biome, revealed that bioclimatic factors at this spatial scale were more limiting for the ranges of *Peltigera* species than the availability of their *Nostoc* partners (Lu et al., 2018).

Modes of reproduction can drive variation in specificity because it translates directly into how photobionts are transmitted from one generation to the next (Otálora et al., 2010; Dal Grande et al., 2012; Hestmark et al., 2016). We looked for signatures of sexual and asexual reproduction by examining whether there was a correlation between the presence of sexual structures (apothecia) on lichen thalli of sections *Peltidea* and *Chloropeltigera*, and their ITS haplotype diversity. Although our molecular sampling was not designed to test directly the prevalence of different types of reproduction, we found a significant positive correlation between the frequency of occurrence of apothecia and ITS haplotype diversity of the mycobiont (Figure 5B). We used these two variables as a proxy to assess the possible role of reproductive modes in shaping the observed specificity patterns. We found that both the ITS haplotype diversity and the frequency of apothecia were higher in section *Chloropeltigera* than in section *Peltidea* (Figures 5C–D). We expect that asexually reproducing species (involving vertical transmission of photobionts) will display specialist patterns of association with their photobionts even if they do not have specialized vegetative propagules (such as soredia and isidia) containing the mycobiont and photobiont, but instead can disperse through thallus fragments, whereas sexually reproducing species (horizontal transmission of photobiont) are more likely to be generalists. The role of asexual reproduction in the specificity of association toward *Nostoc* has been shown for a species pair in *Degelia* (Otálora et al., 2013) as well as for *Collema* and *Leptogium* species with contrasting modes of reproduction (Otálora et al., 2010). Magain et al. (2017a) also observed a trend for higher level of specificity in predominantly asexual species of *Peltigera* in section *Polydactylon.* However, when this hypothesis was tested in bimembered lichen-forming fungi associated with green algae, their reproductive mode was not a good predictor of the level of specificity (Wornik and Grube, 2010). Our findings for trimembered lichens are in agreement with conclusions based on bimembered cyanolichens and lichens associated solely with green algae, because asexual reproduction seems to increase the specificity toward *Nostoc* but not toward *Coccomyxa* (Figure 4). It has been hypothesized that symbiotic interactions can trigger switches in reproductive mode (Buschbom and Mueller, 2006); however, the specific conditions promoting these selective sweeps remain unknown. These observations and predictions should be further addressed in studies specifically designed to determine the factors driving sexual vs. asexual reproduction in lichen-forming fungi, and the evolutionary and genetic mechanisms shaping specificity levels.

## Author Contributions

FL, JM, NM, TG, and CP-D conceived the study. FL, JM, TG, and NM collected the samples. CP-D and NM obtained the molecular data. CP-D performed the phylogenetic and statistical analyses. CP-D, NM, FL, TG, SR, and JM wrote the manuscript.

## Conflict of Interest Statement

The authors declare that the research was conducted in the absence of any commercial or financial relationships that could be construed as a potential conflict of interest.

## Footnotes

1 http://mafft.cbrc.jp/alignment/server/

2 http://purl.org/phylo/treebase/phylows/study/TB2:S23188

3 http://species.h-its.org/
