# Supplementary material for: Contrasting Symbiotic Patterns in Two Closely Related Lineages of Trimembered Lichens of the Genus Peltigera
Source: Front Microbiol. 2018 Nov 16;9:2770. doi: 10.3389/fmicb.2018.02770 (PMC6250826; doi:10.3389/fmicb.2018.02770)
Supplement: Supplementary file 1 [file Data_Sheet_1.DOCX]

Supplementary Material

**Contrasting symbiotic patterns in two sister lineages of trimembered lichens of the genus *Peltigera.***

Carlos José Pardo-De la Hoz*, Nicolas Magain, François Lutzoni, Trevor Goward, Silvia Restrepo, Jolanta Miadlikowska

*** Correspondence:** Carlos José Pardo-De la Hoz

# Supplementary Data





**Supplementary Figure S1.** Maximum likelihood phylogeny based on the six-locus combined mycobiont dataset including 42 specimens from *Peltigera*, section *Chloropeltigera.* Representatives of sections *Phlebia* and *Peltidea* were used as an outgroup (Magain et al. 2017a). Numbers above branches are bootstrap values, and thick branches represent nodes with bootstrap values $\geq$ 70%.

Supplementary Table S1. Taxon sampling with associated voucher information or published source; including mycobiont (*Peltigera*), cyanobiont (*Nostoc*), and phycobiont (*Coccomyxa*) GenBank accession numbers for each specimen used in this study. When applicable, the cyanobiont *rbcLX* phylogroup (roman numbering) and/or haplotype (number preceded by HT) is specified. *CHL: Chloropeltigera; PHL: Phlebia; PLT: Peltidea*; ALA: Alaska; ALB: Alberta; ARC: Arctic; CE: Central Europe; ENA: Eastern North America; MWNA: Mid-Western North America; NE: Northern Europe; PNW: Pacific Northwest; RUS: Russia; SEA: South East Asia; N/A: not applicable; * sequence generated as part of Miadlikowska et al., (unpublished); - no sequence available.

| **DNA id.** | **Species** | **Section** | **Voucher** | **Region** | **ITS** | **β-tubulin** | ***RPB1*** | **COR1b** | **COR3** | **COR16** | **No. of loci** | ***rbcLX* (*Nostoc)*** | **ITS (*Coccomyxa*)** | ***Nostoc* phylogroup or haplotype** | | ***Coccomyxa* species** | | | **Apothecia** |
| --- | --- | --- | --- | --- | --- | --- | --- | --- | --- | --- | --- | --- | --- | --- | --- | --- | --- | --- | --- |
|  |  |  |  |  |  |  |  |  |  |  |  |  |  |  |  | | | |  |
| P1335 | *P. latiloba* | *CHL* | Canada, Nunavik, ULF | ARC | MH734671 | MH756828 | MH756776 | - | MH756981 | - | 4 | MH757036 | - | XVIII | - | | | | N/A |
| P1343 | *P. latiloba* | *CHL* | Canada, Nunavik, ULF 594931 | ARC | MH734633 | MH756830 | MH756760 | - | MH756989 | - | 4 | MH757035 | - | XVIII | - | | | | N/A |
| P1349 | *P. latiloba* | *CHL* | Canada, Nunavik, ULF 594777 | ARC | MH734632 | MH756829 | MH756761 | - | MH756982 | - | 4 | MH757034 | MH753191 | XVIII | *C. solorinae* | | | | Absent |
| P1351 | *P. latiloba* | *CHL* | Canada, Nunavik, ULF 594943 | ARC | MH734631 | MH756827 | MH756775 | - | MH756978 | - | 4 | - | - | - | - | | | | Absent |
| P6080 | *P. latiloba* | *CHL* | Canada, Nunavut, C. Bjork s.n., DUKE | ARC | MH734668 | MH756856 | - | MH756888 | MH756990 | MH756923 | 5 | MH757008 | MH753198 | VI | *C. solorinae* | | | | Absent |
| P6081 | *P. latiloba* | *CHL* | U.S., Alaska, P. Nelson s.n. | ALA | MH734669 | MH756871 | - | - | - | - | 2 | MH757033 | MH753201 | XXV | *C. solorinae* | | | | Absent |
| P702 | *P. leucophlebia 1* | *CHL* | U.S., Alaska, P. Nelson s.n., DUKE | ALA | MH734645 | MH756834 | - | - | MH756991 | - | 3 | - | MH753230 | - | *C. solorinae* | | | | Absent |
| P1036 | *P. leucophlebia 1* | *CHL* | U.S., Vermont, NYBG 1217957 | ENA | MH734647 | MH756846 | MH756755 | - | MH756977 | - | 4 | - | MH753190 | - | *C. solorinae* | | | | Absent |
| P250 | *P. leucophlebia 1* | *CHL* | Canada, British Columbia, T. Goward s.n., UBC 11-040 | PNW | MH734679 | - | MH756765 | - | MH756942 | - | 3 | MH757031 | MH733944 | XXXa | *C. subellipsoidea* | | | | Present |
| P263 | *P. leucophlebia 1* | *CHL* | Canada, British Columbia, T. Goward 10-064, UBC 11-053 | PNW | MH734680 | - | MH756772 | - | MH756943 | - | 3 | - | - | - | - | | | | Present |
| P264 | *P. leucophlebia 1* | *CHL* | Canada, British Columbia, T. Goward 09-107, UBC 11-054 | PNW | MH734681 | - | MH756758 | - | MH756936 | - | 3 | MH757027 | MH753180 | VI | *C. subellipsoidea* | | | | Present |
| P254 | *P. leucophlebia 1* | *CHL* | Canada, British Columbia, T. Goward 09-435, UBC 11-044 | PNW | MH734682 | - | - | - | MH756937 | - | 2 | MH757028 | - | H466 | - | | | | Present |
| P251 | *P. leucophlebia 1* | *CHL* | Canada, British Columbia, T. Goward 09 s.n., UBC 11-041 | PNW | MH734683 | - | - | - | MH756938 | - | 2 | - | - | - | - | | | | Present |
| P283 | *P. leucophlebia 1* | *CHL* | Canada, British Columbia, UBC 11-082 | PNW | MH734684 | - | - | - | MH756939 | - | 2 | MH757020 | - | V | - | | | | Absent |
| P257 | *P. leucophlebia 1* | *CHL* | Canada, British Columbia, T. Goward 09-292, UBC 11-047 | PNW | MH734685 | - | MH756773 | - | MH756940 | - | 3 | - | - | - | - | | | | Absent |
| P1371a | *P. leucophlebia 1.* | *CHL* | Russia, Murmansk, DUKE | NE | MH734686 | MH756816 | - | - | MH756941 | - | 3 | - | - | - | - | | | | Absent |
| P6061 | *P. leucophlebia 1* | *CHL* | U.S., Alaska, Spickerman, McCune, Nelson, Tonsberg & Walton s.n., DUKE | ALA | MH734687 | MH756863 | MH756780 | MH756891 | MH756949 | MH756909 | 6 | MH757038 | MH753192 | XLII | *C. solorinae* | | | | Absent |
| P6072 | *P. leucophlebia 1* | *CHL* | Russia, Khabarovsk, J. Miadlikowska s.n., DUKE | SEA | MH734688 | MH756852 | MH756788 | MH756895 | MH756987 | MH756901 | 6 |  | MH753207 |  | *C. solorinae* | | | | Absent |
| P1371b | *P. leucophlebia 1.* | *CHL* | Russia, Murmansk, DUKE | NE | MH734689 | MH756817 | - | - | MH756988 | - | 3 | - | - | - | - | | | | Absent |
| P6059 | *P. leucophlebia 1* | *CHL* | U.S., Alaska, Spickerman, McCune, Nelson, Tonsberg & Walton 174, DUKE | ALA | MH734690 | MH756851 | MH756797 | MH756890 | MH756993 | MH756903 | 6 | MH757013 | MH753170 | XVIII | *C. subellipsoidea* | | | | Absent |
| P262 | *P. leucophlebia 1* | *CHL* | Canada, British Columbia, T. Goward 09-634, UBC 11052 | PNW | MH734691 | - | - | - | MH756992 | - | 2 | - | - | - | - | | | | Present |
| P239 | *P. leucophlebia 1* | *CHL* | Canada, British Columbia, T. Goward 07-079, UBC 11-025 | PNW | MH734692 | MH756847 | MH756771 | - | MH756984 | - | 4 | MH757042 | - | VI | - | | | | Absent |
| P259 | *P. leucophlebia 1* | *CHL* | Canada, British columbia, T. Goward 09-633, UBC 11-049 | PNW | MH734693 | - | MH756767 | - | MH756985 | - | 3 | - | - | - | - | | | | Present |
| P6050 | *P. leucophlebia 1* | *CHL* | Austria, Styria, J. Miadlikowska & Hafellner s.n., DUKE 38906 | CE | MH734694 | - | - | - | MH756986 | - | 2 | MH757004 | MH753229 | VI | *C. solorinae* | | | | Absent |
| P6065 | *P. leucophlebia 1* | *CHL* | Iceland, Arnessysla, J. Miadlikowska & F. Lutzoni s.n., DUKE | ARC | MH734695 | MH756812 | MH756783 | MH756889 | MH756944 | MH756900 | 6 | MH757054 | MH753164 | XVI | *C. subellipsoidea* | | | | Absent |
| P6076 | *P. leucophlebia 1* | *CHL* | Canada, British Columbia, T. Goward 14-001, DUKE | PNW | MH734696 | - | - | MH756899 | MH756947 | - | 3 | MH757045 | MH753159 | VI | *C. subellipsoidea* | | | | Absent |
| P6091 | *P. leucophlebia 1* | *CHL* | Canada, Alberta, J. Miadlikowska s.n., DUKE | ALB | MH734697 | MH756864 | - | - | - | MH756902 | 3 | MH757016 | MH753208 | XXXIIIa | *C. solorinae* | | | | Present |
| P6075 | *P. leucophlebia 1.* | *CHL* | U.S., Wyoming, J. Hollinger 2593, DUKE | MWNA | MH734698 | - | - | MH756898 | MH756948 | - | 3 | MH757029 | MH753167 | XXXa | *C. subellipsoidea* | | | | Absent |
| P1118 | *P. leucophlebia 2* | *CHL* | U.S., South Dakota, DUKE | MWNA | MH734627 | MH756818 | - | - | MH756945 | - | 3 | MH757025 | MH753225 | VI | *C. solorinae* | | | | Present |
| P1119 | *P. leucophlebia 2* | *CHL* | U.S., South Dakota, DUKE | MWNA | MH734628 | - | - | - | MH756946 | - | 2 | - | - | - | - | | | | Present |
| P1355 | *P. leucophlebia 2* | *CHL* | Finland, Sysma, H 26615 | NE | MH734629 | MH756815 | MH756768 | - | MH756994 | - | 4 | - | - | - | - | | | | Present |
| P1120 | *P. leucophlebia 2* | *CHL* | Canada, Alberta, DUKE | ALB | MH734630 | MH756819 | - | - | MH756995 | - | 3 | - | - | - | - | | | | Absent |
| P246 | *P. leucophlebia 2* | *CHL* | Canada, British Columbia, T. Goward 07-193, UBC 11-032 | PNW | MH734634 | MH756831 | MH756769 | - | MH756950 | - | 4 | - | - | - | - | | | | Absent |
| P288 | *P. leucophlebia 2* | *CHL* | Canada, British Columbia, Talbot 002-23,UBC 11-089 | PNW | MH734639 | MH756824 | - | - | MH756958 | - | 3 | MH757056 | MH753182 | VI | *C. subellipsoidea* | | | | Present |
| P289 | *P. leucophlebia 2* | *CHL* | Canada, British Columbia, T. Goward 09-115, UBC 11-090 | PNW | MH734638 | MH756822 | - | - | MH756959 | - | 3 | - | MH753177 | - | *C. subellipsoidea* | | | | Present |
| P710 | *P. leucophlebia 2* | *CHL* | U.S., Alaska, P. Nelson s.n., DUKE | ALA | MH734640 | MH756825 | - | - | MH756956 | - | 3 | - | - | - | - | | | | Absent |
| P711 | *P. leucophlebia 2* | *CHL* | U.S., Alaska, P. Nelson s.n., DUKE | ALA | MH734636 | MH756820 | - | - | MH756957 | - | 3 | - | MH753181 | - | *C. subellipsoidea* | | | | Present |
| P716 | *P. leucophlebia 2* | *CHL* | U.S., Alaska, P. Nelson s.n., DUKE 7808 | ALA | MH734635 | MH756832 | - | - | MH756954 | - | 3 | - | MH753178 | - | *C. subellipsoidea* | | | | Absent |
| P286 | *P. leucophlebia 2* | *CHL* | Canada, British columbia, T. Goward 09-097, UBC 11-087 | PNW | MH734637 | MH756821 | - | - | MH756955 | - | 3 | MH757049 | MH753172 | VI | *C. subellipsoidea* | | | | Present |
| P762 | *P. leucophlebia 2* | *CHL* | Canada, British Columbia, H 68797 | PNW | MH734642 | MH756873 | - | - | MH756953 | - | 3 | - | - | - | - | | | | Absent |
| P287 | *P. leucophlebia 2* | *CHL* | Canada, British Columbia, T. Goward 10-093, UBC 11-088 | PNW | MH734643 | MH756826 | - | - | MH756952 | - | 3 | MH757044 | MH753186 | VI | *C. subellipsoidea* | | | | Absent |
| P704 | *P. leucophlebia 2* | *CHL* | U.S., Alaska, P. Nelson s.n., DUKE | ALA | MH734644 | MH756869 | - | - | MH756951 | - | 3 | - | - | - | - | | | | N/A |
| P705 | *P. leucophlebia 2* | *CHL* | U.S., Alaska, P. Nelson s.n., DUKE | ALA | MH734646 | MH756833 | MH7567775 | - | MH756976 | - | 4 | MH757055 | MH753232 | XVIII | *C. solorinae* | | | | Absent |
| P6068 | *P. leucophlebia 2* | *CHL* | China, B. Goffinet | SEA | MH734658 | MH756861 | MH756785 | MH756894 | MH756961 | MH756924 | 6 | - | MH753210 | - | *C. solorinae* | | | | Absent |
| P6086 | *P. leucophlebia 2* | *CHL* | Canada, British Columbia, T. Goward 15-029, DUKE | PNW | MH734659 | - | - | - | - | MH756921 | 2 | - | MH753206 | - | *C. solorinae* | | | | Present |
| P6085 | *P. leucophlebia 2* | *CHL* | Canada, Alberta, J. Miadlikowska s.n., DUKE | ALB | MH734660 | MH756858 | MH778710 | - | - | MH756908 | 4 | MH757048 | MH753199 | XXXIIIa | *C. solorinae* | | | | Present |
| P6087 | *P. leucophlebia 2* | *CHL* | Canada, British Columbia, T. Goward 15xxx029, DUKE | PNW | MH734661 | - | MH778711 | - | - | MH756914 | 3 | - | MH753212 | - | *C. solorinae* | | | | Present |
| P6060 | *P. leucophlebia 2* | *CHL* | U.S., Alaska, Spickerman, McCune, Nelson, Tonsberg & Walton s.n., DUKE | ALA | MH734662 | MH756853 | MH756798 | MH756896 | MH756983 | MH756904 | 6 | - | MH753226 | - | *C. solorinae* | | | | Present |
| P6083 | *P. leucophlebia 2* | *CHL* | Canada, Alberta, J. Miadlikowska s.n., DUKE | ALB | MH734663 | MH756857 | MH756791 | MH756893 | MH756960 | MH756905 | 6 | MH757010 | MH753165 | VI | *C. subellipsoidea* | | | | Absent |
| P6079 | *P. leucophlebia 2* | *CHL* | Canada, British Columbia, T. Goward 15-030 DUKE | PNW | MH734664 | - | - | - | - | - | 1 | MH757037 | MH753169 | XXXa | *C. subellipsoidea* | | | | Absent |
| P6078 | *P. leucophlebia 2* | *CHL* | Canada, Alberta, T. Goward 12-278, DUKE | PNW | MH734665 | - | - | - | - | MH756925 | 2 | MH757015 | MH753197 | XXXIIIa | *C. solorinae* | | | | Absent |
| P6090 | *P. leucophlebia 2* | *CHL* | Canada, Ontario, T. McMullin 8924, DUKE | ENA | MH734666 | MH756859 | - | - | - | - | 2 | MH757019 | MH753155 | V | *C. subellipsoidea* | | | | Absent |
| P6092 | *P. leucophlebia 2* | *CHL* | Canada, Quebec, J. Miadlikowska s.n., DUKE | ENA | MH734670 | MH756860 | MH756802 | MH756892 | - | - | 4 | MH757017 | MH753196 | XXXa | *C. solorinae* | | | | Absent |
| P243 | *P. leucophlebia 3* | *CHL* | Canada, British Columbia, T. Goward s.n., UBC 11-029 | PNW | MH734648 | MH756840 | MH756793 | - | MH756974 | - | 4 | - | - | - | - | | | | N/A |
| P1068 | *P. leucophlebia 3* | *CHL* | U.S., Wyoming, MIN 899752 | MWNA | MH734649 | MH756838 | MH756757 | - | MH756964 | - | 4 | - | - | - | - | | | | Present |
| P241 | *P. leucophlebia 3* | *CHL* | Canada, British Columbia, T. Goward 09-113, UBC 11-027 | PNW | MH734650 | MH756842 | MH756763 | - | MH756971 | - | 4 | MH757030 | MH753156 | XXXa | *C. subellipsoidea* | | | | Present |
| P253 | *P. leucophlebia 3* | *CHL* | Canada, British Columbia, T. Goward 06-1530b, UBC 11-043 | PNW | MH734651 | MH756843 | MH756766 | - | MH756970 | - | 4 | MH757043 | MH753179 | VI | *C. subellipsoidea* | | | | Present |
| P240 | *P. leucophlebia 3* | *CHL* | Canada, British Columbia, T. Goward 06-1529a, UBC 11-026 | PNW | MH734652 | MH756841 | MH756764 | - | MH756972 | - | 4 | MH757046 | MH753176 | VI | *C. subellipsoidea* | | | | Absent |
| P242 | *P. leucophlebia 3* | *CHL* | Canada, British Columbia, T. Goward 09-118, UBC 11-028 | PNW | MH734654 | MH756844 | MH756770 | - | MH756975 | - | 4 | - | - | - | - | | | | Present |
| P270 | *P. leucophlebia 3* | *CHL* | Canada, British Columbia, T. Goward 09-130, UBC 11-062 | PNW | MH734655 | MH756845 | MH756759 | - | MH756969 | - | 4 | MH757024 | - | VI | - | | | | Present |
| P238 | *P. leucophlebia 3* | *CHL* | Canada, British Columbia, T. Goward 09-125, UBC 11-024 | PNW | MH734656 | MH756839 | MH756762 | - | MH756973 | - | 4 | MH757023 | MH753175 | VI | *C. subellipsoidea* | | | | Present |
| P6056 | *P. leucophlebia 3* | *CHL* | U.S., California, James R. Shevock 25900, NYBG | PNW | MH734657 | - | - | - | - | - | 1 | - | MH753158 | - | *C. subellipsoidea* | | | | Absent |
| P6066 | *P. leucophlebia 3* | *CHL* | U.S., Colorado, DUKE | MWNA | MH734667 | MH756872 | MH756784 | - | MH756965 | MH756911 | 5 | MH757009 | MH753153; MH753152 | VI | *C. subellipsoidea* | | | | Present |
| P1082 | *P. leucophlebia 3* | *CHL* | U.S., New Mexico, DUKE 2465 | MWNA | MH734653 | - | MH756794 | - | MH756967 | - | 3 | MH757052 | MH753184 | V | *C. subellipsoidea* | | | | Absent |
| P258 | *P. leucophlebia 4* | *CHL* | Canada, British Columbia, T. Goward 07-052, UBC 11-048 | PNW | MH734672 | MH756835 | - | - | MH756968 | - | 3 | MH757032 | MH753163 | XXXa | *C. subellipsoidea* | | | | Present |
| P1071 | *P. leucophlebia 4* | *CHL* | U.S., Wyoming, MIN 898865 | MWNA | MH734673 | - | MH756756 | - | MH756966 | - | 3 | - | - | - | - | | | | Absent |
| P245 | *P. leucophlebia 4* | *CHL* | Canada, British Columbia, T. Goward 07-054, UBC 11-031 | PNW | MH734674 | MH756836 | MH756774 | - | MH756962 | - | 4 | - | MH753231 | - | *C. solorinae* | | | | Present |
| P267 | *P. leucophlebia 4* | *CHL* | Canada, British Columbia, T. Goward 09-631, UBC 11-059 | PNW | MH734675 | MH756837 | - | - | MH756963 | - | 3 | MH757026 | MH753183 | VI | *C. subellipsoidea* | | | | Absent |
| P6074 | *P. leucophlebia 4* | *CHL* | U.S., Wyoming, R. Harms 1419, DUKE 313518 | MWNA | MH734676 | - | - | - | - | - | 1 | MH757011 | MH753168 | VI | *C. subellipsoidea* | | | | Absent |
| P6077 | *P. leucophlebia 4* | *CHL* | Canada, British Columbia, T. Goward 15-029, DUKE | PNW | MH734677 | MH756855 | - | - | - | MH756906 | 3 | MH757041 | MH753166 | VI | *C. subellipsoidea* | | | | Absent |
| P6064 | *P. leucophlebia 4* | *CHL* | Iceland, Langisjór, Starri Heidmarsson 1983, DUKE | ARC | MH734678 | MH756854 | MH756782 | - | MH756979 | MH756910 | 5 | MH757040 | MH753205 | VI | *C. solorinae* | | | | Absent |
| P1354 | *P. leucophlebia 6* | *CHL* | Switzerland, Kanton Glarus, H 23801 | CE | MH734699 | MH756813 | MH756778 | - | - | - | 3 | - | - | - | - | | | | Present |
| P1353 | *P. leucophlebia 6* | *CHL* | France, Languedoc-Rousillon, DUKE | CE | MH734700 | MH756814 | - | - | MH756980 | - | 3 | - | - | - | - | | | | Absent |
| P6052 | *P. leucophlebia 6* | *CHL* | Austria, Styria, J. Miadlikowska & Hafellner s.n., DUKE 38929 | CE | MH734701 | - | - | - | - | - | 1 | MH757007 | MH753227 | VI | *C. solorinae* | | | | Absent |
| P6055 | *P. leucophlebia 6* | *CHL* | Austria, Styria, J. Miadlikowska & Hafellner | CE | MH734702 | MH756849 | - | - | MH756928 | MH756922 | 4 | - | MH753228 | - | *C. solorinae* | | | | Absent |
| P1056 | *P. leucophlebia 6* | *CHL* | U.S., Michigan, MIN 882969 | ENA | MH734703 | - | MH756753 | - | - | - | 2 | - | - | - | - | | | | Absent |
| P1057 | *P. leucophlebia 6* | *CHL* | U.S., Michigan, MIN 882779 | ENA | MH734704 | - | MH756754 | - | - | - | 2 | - | - | - | - | | | | Present |
| P6062 | *P. leucophlebia 6* | *CHL* | Iceland, Arnessysla, J. Miadlikowska & F. Lutzoni s.n., DUKE | ARC | MH734705 | MH756850 | MH756781 | MH756897 | MH756933 | MH756912 | 6 | MH757039 | MH753193 | VI | *C. solorinae* | | | | Absent |
| P6063 | *P. leucophlebia 6* | *CHL* | Iceland, Arnessysla, J. Miadlikowska & F. Lutzoni s.n., DUKE | ARC | MH734706 | MH756870 | MH756799 | MH756881 | MH756932 | MH756915 | 6 | MH757005 | MH753194 | XLI | *C. solorinae* | | | | Present |
| P6088 | *P. leucophlebia 6* | *CHL* | Canada, Nova Scotia, T. McMullin 7989, DUKE | ENA | MH734707 | - | MH756801 | - | - | MH756907 | 3 | MH757006 | MH753200 | H125 | *C. solorinae* | | | | Absent |
| P6082 | *P. leucophlebia 6* | *CHL* | Canada, Alberta, J. Miadlikowska s.n., DUKE | ALB | MH734708 | MH756868 | MH756790 | - | MH756998 | MH756918 | 5 | MH757018 | MH753203 | XXXIIIa | *C. solorinae* | | | | Absent |
| P6084 | *P. leucophlebia 6* | *CHL* | Canada, Alberta, J. Miadlikowska s.n., DUKE | ALB | MH734726 | - | MH756792 | MH756882 | - | - | 3 | - | - | - | - | | | | Absent |
| O41493 | *P. nigripunctata s. str.* | *CHL* | South Korea, HUR 041493 | SEA | MH734716 | MH756810 | MH756779 | - | MH756927 | MH756926 | 5 | - | MH753154; MH753151 | - | *C. solorinae* | | | | Absent |
| P1087 | *P. nigripunctata s. str.* | *CHL* | China, SW Sichuan, H 47470 | SEA | MH734717 | - | MH756795 | - | - | - | 2 | - | - | - | - | | | | Present |
| P6002 | *P. nigripunctata s. str.* | *CHL* | Japan, Hokkaido, T. Tonsberg TT 23216, CONN | SEA | MH734715 | - | - | - | - | - | - | - | MH753213 | - | *C. solorinae* | | | | Absent |
| P1133 | *P. leucophlebia 5* | *CHL* | U.S., Alaska, DUKE | ALA | MH734709 | MH756848 | - | - | MH756996 | - | 3 | - | - | - | - | | | | Absent |
| P290 | *P. leucophlebia 5* | *CHL* | Canada, British Columbia, T. Goward 09-108, UBC 11-091 | PNW | MH734641 | MH756823 | - | - | MH756997 | - | 3 | MH757050 | - | VI | - | | | | Absent |
| P6058 | *P. leucophlebia 5* | *CHL* | U.S., Alaska, J K Walton 18638, DUKE | ALA | MH734710 | MH756865 | MH756796 | MH756886 | MH756929 | MH756919 | 6 | MH757021 | MH753171 | V | *C. subellipsoidea* | | | | Present |
| P6067 | *P. leucophlebia 5* | *CHL* | China, B. Goffinet | SEA | MH734711 | MH756862 | MH756800 | MH756883 | MH756934 | MH756917 | 6 | - | MH753209 | - | *C. solorinae* | | | | Absent |
| P6069 | *P. leucophlebia 5* | *CHL* | Russia, Khabarovsk, J. Miadlikowska s.n., DUKE | SEA | MH734714 | MH756811 | MH756786 | MH756887 | MH756931 | MH756913 | 6 | - | MH753211 | - | *C. solorinae* | | | | Present |
| P6070 | *P. leucophlebia 5* | *CHL* | Russia, Khabarovsk, J. Miadlikowska s.n., DUKE | SEA | MH734712 | MH756866 | MH756787 | MH756885 | MH756930 | MH756920 | 6 | MH757022 | MH753195 | V | *C. solorinae* | | | | Absent |
| P6073 | *P. leucophlebia 5* | *CHL* | Russia, Khabarovsk, J. Miadlikowska s.n., DUKE | SEA | MH734713 | MH756867 | MH756789 | MH756884 | MH756935 | MH756916 | 6 | MH757014 | MH753202 | H144 | *C. solorinae* | | | | Absent |
| P1330 | *P. aphthosa* 1 | *PLT* | Canada, Québec, Nunavik, L. Couillard s.n., QFA-0594935 | ENA | - | - | - | - | - | - | 0 | MH757051 | MH753221 | V | *C. solorinae* | | | N/A | |
| P1332 | *P. aphthosa* 1 | *PLT* | Canada, Québec, Nunavik, J. Gagnon s.n., QFA-0595018 | ENA | - | - | - | - | - | - | 0 | MH757057 | MH753150 | VI | *C. solorinae* | | | Absent | |
| P1312 | *P. aphthosa* 2 | *PLT* | U.S., Alaska, Nome, J. Miadlikowska et al. s.n., 06. 2008, DUKE | ALA | - | - | - | - | - | - | 0 | MH757073* | MH753218 | IV | *C. solorinae* | | | Absent | |
| P1309 | *P. aphthosa* 2 | *PLT* | U.S., Alaska, Nome, J. Miadlikowska et al. s.n., 06. 2008, DUKE | ALA | - | - | - | - | - | - | 0 | MH757012 | MH753217 | VI | *C. solorinae* | | | Absent | |
| P776 | *P. aphthosa* 3 | *PLT* | Russia, Kamchatka, D. E. Himelbrant s.n., 03.08.2009, H | RUS | - | - | - | - | - | - | 0 | MH757070* | MH753223 | IV | *C. solorinae* | | | Present | |
| P4025 | *P. aphthosa* 3 | *PLT* | Canada, Québec, R. Darnajoux, 06.2013, Bms4-39, DUKE | ENA | - | - | - | - | - | - | 0 | MH757084* | MH753187 | IV | *C. subellipsoidea* | | | Absent | |
| P4043 | *P. aphthosa* 4 | *PLT* | U.S., Alaska, K. Spickerman s.n., 08.01.2013, DUKE | ALA | MH734723 | MH756806 | - | MH756878 | - | - | 0 | MH757066* | - | IV | - | |  | | Absent |
| P790 | *P. aphthosa* 4 | *PLT* | Norway, N. Magain s.n., 2011, LG | NE | - | - | - | - | - | - | 0 | MH757071* | MH753216 | IV | *C. solorinae* | | | Absent | |
| P1184 | *P. aphthosa* 4 | *PLT* | Finland, B. Krzewicka 2512, UPS L-153684 | NE | - | - | - | - | - | - | 0 | MH757072* | MH753224 | IV | *C. solorinae* | | | Absent | |
| P774 | *P. aphthosa* 5 | *PLT* | Russia, Krasnoyarskiy kray, V. Pervunin s.n., 07. 2000, H | RUS | - | - | - | - | - | - | 0 | MH757062* | - | IV | - | | | | Present |
| P1172 | *P. aphthosa* 5 | *PLT* | U.S., Minnesota, C. Wetmore 92428, MIN | ENA | - | - | - | - | - | - | 0 | MH757063* | - | IV | - | | | | Absent |
| P4048 | *P. aphthosa* 5 | *PLT* | Canada, Ontario, R. Troy McMullin 8928, DUKE | ENA | - | - | - | - | - | - | 0 | MH757064* | MH753215 | IV | *C. solorinae* | | | Absent | |
| P725 | *P. aphthosa* 5 | *PLT* | Canada, Québec W6C1, J. Miadlikowska et al. s.n., DUKE | ENA | - | - | - | - | - | - | 0 | MH757065* | - | IV | - | |  | | Absent |
| P4049 | *P. aphthosa* 5 | *PLT* | Canada, Ontario, R. Troy McMullin 8920, DUKE | ENA | - | - | - | - | - | - | 0 | MH757067* | MH753222 | IV | *C. solorinae* | | | Absent | |
| P1189 | *P. aphthosa* 5 | *PLT* | Sweden, I. Karlsson s.n., 10.23.2011, UPS L-552785 | NE | - | - | - | - | - | - | 0 | MH757068* | MH753220 | IV | *C. solorinae* | | | Absent | |
| P709 | *P. aphthosa* 5 | *PLT* | U.S., Alaska, J. Miadlikowska and F. Lutzoni s.n., 08.09.2011, DUKE | ALA | - | - | - | - | - | - | 0 | MH757069* | MH753189 | IV | *C. solorinae* | | | Absent | |
| P726 | *P. aphthosa* 5 | *PLT* | Canada, Québec W50C3, J. Miadlikowska et al. s.n., DUKE | ENA | - | - | - | - | - | - | 0 | MH757077* | MH753162 | IV | *C. subellipsoidea* | | | Absent | |
| P708 | *P. aphthosa* 5 | *PLT* | U.S., Alaska, J. Miadlikowska and F. Lutzoni s.n., 08.09.2011, DUKE | ALA | - | - | - | - | - | - | 0 | MH757081* | MH753188 | IV | *C. solorinae* | | | Absent | |
| P1114 | *P. aphthosa* 5 | *PLT* | Canada, Alberta, J. Hollinger 3870, UBC | ALB | - | - | - | - | - | - | 0 | MH757047 | MH753219 | VI | *C. solorinae* | | | Absent | |
| P1324 | *P. britannica* 1 | *PLT* | Canada, British Columbia, J. Lendemer 22342, NYBG 01221288 | PNW | - | - | - | - | - | - | 0 | MH757060* | MH753160 | IV | *C. subellipsoidea* | | | Present | |
| P1066 | *P. britannica* 2 | *PLT* | U.S., Wyoming, C. Wetmore 96597, MIN | MWNA | - | - | - | - | - | - | 0 | MH757053* | MH753185 | VI | *C. subellipsoidea* | | | Present | |
| P237 | *P. chionophila* | *PLT* | Canada, British ColombiaC, T. Goward 06-1255, UBC | PNW | MH734719 | MH756803 | - | MH756874 | MH756999 | - | 0 | MH757058* | MH733943 | IV | *C. subellipsoidea* | | | Absent | |
| P4039 | *P. chionophila* | *PLT* | Canada, British Columbia, Björk 19447, UBC | PNW | - | - | - | - | - | - | 0 | MH757059* | MH753161 | IV | *C. subellipsoidea* | | | N/A | |
| P252 | *P. chionophila* | *PLT* | Canada, British Columbia, T. Goward 06-1375, UBC | PNW | - | - | - | - | - | - | 0 | MH757061* | - | IV | - | | | | Absent |
| P1142 | *P. chionophila* | *PLT* | Canada, BC, T. Goward 02-414, UBC | PNW | - | - | - | - | - | - | 0 | MH757074* | MH753157 | IV | *C. subellipsoidea* | | | Present | |
| P1140 | *P. chionophila* | *PLT* | Canada, BC, T. Goward 02-398, UBC | PNW | - | - | - | - | - | - | 0 | MH757075* | MH753174 | IV | *C. subellipsoidea* | | | Absent | |
| P4041 | *P. chionophila* | *PLT* | Canada, BC, T. Goward 95-15, UBC | PNW | - | - | - | - | - | - | 0 | MH757078* | - | IV | - | |  | | Absent |
| P1174 | *P. frippii* | *PLT* | Norway, Finmark, O. Vitikainen 8323, H | NE | - | - | - | - | - | - | 0 | MH757079* | - | IV | - | |  | | Absent |
| P4036 | *P. frippii* | *PLT* | Canada, Nunavut, C. Björk 27565, UBC | ARC | MH734720 | MH756807 | - | MH756875 | - | - | 0 | MH757080* | - | IV | - | |  | | N/A |
| P4037 | *P. frippii* | *PLT* | Canada, Nunavut, C. Björk 27420, UBC | ARC | - | - | - | - | - | - | 0 | MH757082* | - | IV | - | |  | | N/A |
| P1180 | *P. malacea* 1 | *PLT* | Russia, Sakha Republic, T. Ahti 61978, H | RUS | - | - | - | - | - | - | 0 | MH757083* | - | III | - | |  | | Absent |
| P1129 | *P. malacea* 5 | *PLT* | Canada, Alberta, J. Hollinger 3830, UBC | ALB | - | - | - | - | - | - | 0 | MH757076* | MH753214 | IV | *-* | |  | | Absent |
| P6094 | *P. venosa* | *PHL* | U.S., Utah, C.Truong 4008, DUKE | MWNA | MH734718 | - | - | - | - | - | 0 | MH757003 | MH753204 | XLIV | *C. solorinae* | | | N/A | |
